# Comparative effects of cigarette smoking, e-cigarette use, and dual use on pulmonary function, exercise capacity, and quality of life in young adults: a cross-sectional study

**DOI:** 10.3389/fpubh.2025.1707230

**Published:** 2026-01-14

**Authors:** Gokul G. Krishna, Ann M. Jose, Weaam A. Rahali, Wejdan W. Alyamani, Manahel A. Mohammed, Basmah S. Alghamdi, Mazen M. Homoud, Mohammed Almeshari, Khalid S. Alwadeai, Saleh R. Alkhathami, Jithin K. Sreedharan, Ayedh D. Alahmari

**Affiliations:** 1 Department of Respiratory Therapy, Batterjee Medical College, Jeddah, Saudi Arabia; 2 Department of Respiratory Therapy, Faculty of Medical Rehabilitation Sciences, King Abdulaziz University, Jeddah, Saudi Arabia; 3 Department of Rehabilitation Health Sciences, College of Applied Medical Sciences, King Saud University, Riyadh, Saudi Arabia; 4 Department of Inflammation and Ageing, University of Birmingham, Birmingham, United Kingdom; 5 Department of Medical Laboratory, King Fahad Armed Hospital, Jeddah, Saudi Arabia; 6 Department of Respiratory Therapy, College of Rehabilitation Sciences, University of Manitoba, Winnipeg, MB, Canada

**Keywords:** cigarette smoking, dual use, electronic cigarettes, exercise test, forced expiratory volume, pulmonary function tests, quality of life, six-minute walk test

## Abstract

**Background:**

Tobacco smoking constitutes a primary cause of preventable cardiovascular and pulmonary diseases on a global scale. While smoking rates fall, e-cigarette use rises, especially in youth.

**Objective:**

Assess effects of smoking, vaping, and dual use on lung function, exercise capacity and quality of life.

**Methods:**

Participants were classified into five groups: Control, Cigarette use only, E-cigarettes use only, Ex-cigarette smoker current vaper, and Dual users (use of both e-cigarettes and combustible cigarettes). Participants performed spirometry, six minute walk test (6MWT) and completed health related quality of life (HRQoL) questionnaire.

**Results:**

A total of 222 participants, 86.9% were male, with median age 26 years. Age and body mass index (BMI) showed no significant differences across groups. Cigarette-only and dual users reported 20 cigarettes/day with 5–7 years smoking duration, while e-cigarette use duration was 4–5 years among exclusive vapers, ex-smoker vapers, and dual users. Spirometry revealed impairments: Forced expiratory volume in the first second (FEV_1_; % predicted) was lower across groups versus controls (*H* = 80.69, df = 4, *p* < 0.001, *η*^2^ = 0.35), lowest in dual users. Forced vital capacity (FVC; L) showed no differences, while FVC (% predicted) decreased in smokers and dual users. FEV_1_/FVC ratio was reduced (*H* = 66.54, df = 4, *p* < 0.001, *η*^2^ = 0.29), most in dual users. 6MWT showed no group differences. HRQoL indicated decline in physical functioning (*H* = 35.11, *p* < 0.001, *η*^2^ = 0.14), role limitations due to physical health social functioning, and emotional wellbeing, lowest in cigarette-only and dual users.

**Conclusion:**

Young adults using cigarettes and e-cigarettes showed impaired lung function and quality of life compared to never-users. Daily dual users showed the greatest declines, while former smokers using e-cigarettes showed intermediate outcomes. Exclusive e-cigarette users exhibited least impairment.

## Introduction

Tobacco smoking is a worldwide problem, which is directly linked to chronic obstructive pulmonary disease (COPD), cancer, and cardiovascular disease (1). According to the World Health Organization (WHO), there are approximately 1.3 billion people who smoke tobacco globally, resulting in more than 8 million deaths each year from smoking-associated illnesses. This equates to one death every 6 s attributable to tobacco use (2). Electronic cigarettes (e-cigarettes), introduced in 2003, are a relatively new product on the market (3). E-cigarettes are battery-operated aerosol generators which heat a nicotine-containing solution (e-liquids) compared to burning tobacco such as in conventional cigarette smoking. These e-liquids contain various chemical components, including glycerol or propylene glycol, varying amounts of nicotine and flavorings such as aldehydes, pyrazines, menthol, menthone, and other minty compounds, the practice of using e-cigarettes is commonly known as vaping (4, 5).

In numerous countries, the prevalence of traditional cigarette smoking has decreased, while the use of e-cigarettes has risen across various age groups, particularly among adolescents and young adults (6–12). Recent data from high-income settings indicate that a substantial proportion of current e-cigarette users are former smokers, whereas a significant minority continue to use both products concurrently as dual users (13). Longitudinal and population-based surveys from the United Kingdom and the United States suggest that many individuals transition from smoking to exclusive vaping, yet a persistent segment maintains dual use, raising concerns about ongoing exposure to the toxicants of both products (6, 14). A recent Cochrane review reported high-certainty evidence that nicotine e-cigarettes increase smoking cessation rates compared with nicotine replacement therapy, with similar profiles of common adverse events, supporting their potential role as a cessation aid (15). Concurrently, the growing popularity of e-cigarettes as a perceived safer alternative, along with diverse flavors and greater social acceptability, has contributed to increased uptake among never-smokers, including young adults (11, 16, 17).

The detrimental impact of cigarette smoking is extensively documented, with prolonged use resulting in diminished pulmonary function, impaired exercise capacity, and reduced health-related quality of life due to dyspnea, deconditioning, and comorbidity burden (1). In contrast, the health implications of exclusive vaping and dual use remain less well-defined, particularly among younger populations (18). Existing research on e-cigarette users has yielded heterogeneous results, with some studies indicating reductions in spirometry indices and diffusing capacity of the lung for carbon monoxide (DLCO), while others have reported no significant impairment, often confounded by prior or concurrent smoking and relatively short exposure durations (19, 20). Evidence regarding exercise capacity and health-related quality of life (HRQoL) in exclusive vapers and dual users is even more limited. Emerging data suggest that both smoking and, to a lesser extent, vaping are associated with decreased physical performance and poorer HRQoL, particularly in the physical and mental health domains, with dual users frequently exhibiting the greatest symptom burden (21, 22). However, most available studies focus on older or mixed-age populations, and rarely provide direct comparisons between exclusive smokers, exclusive vapers, dual users, and never-users within the same cohort (7, 23–25).

These evidence gaps highlight the need for direct comparative data on lung function, exercise capacity, and HRQoL among young adults with varying patterns of tobacco and nicotine product use. This cross sectional study therefore aimed to evaluate the pulmonary function, exercise capacity, and quality of life among young adults with daily cigarette smoking, e-cigarette use, or dual use, compared to individuals who have never smoked or vaped.

## Methods

### Participants and assessment

A cross-sectional study was conducted between February 2022 and March 2025 at the Department of Respiratory Therapy, Batterjee Medical College (BMC), Jeddah, Saudi Arabia. Participants who met the inclusion criteria were enrolled and subsequently categorized into five groups according to their cigarette and e-cigarette use status. Demographic information and medical history were obtained at baseline. After baseline assessments, all participants underwent Pulmonary Function Testing (PFT), a Six-Minute Walk Test (6MWT), and completed a validated Health-Related Quality of Life (HRQoL) questionnaire.

### Study protocol

Participants aged ≥18 years were recruited using a multi-modal strategy. Announcements were disseminated through the official communication channels of BMC and supplemented by outreach on social media platforms (Twitter, Facebook, and Snapchat) with digital advertisements highlighting study objectives, eligibility criteria, and contact information. Additional recruitment was conducted *Via* flyers in vape shops and community spaces frequented by smokers and vapers. To maximize participation, recruitment reminders and direct contact options were provided. Eligible participants were allocated into five groups.

1. Control group: individuals who had never smoked cigarettes or used e-cigarettes.
2. Cigarette-only group: daily cigarette smokers with a smoking history of ≥4 pack-years and no history of e-cigarette use (26). Pack year were calculated using standard pack year formula. Pack years = (number of cigarettes smoked per day ÷ 20) × years of smoking.
3. E-cigarette-only group: daily e-cigarette users for ≥4 years with no history of cigarette smoking.
4. Ex-cigarette smoker current vaper group: participants with a history of cigarette smoking but no cigarette use within the past year, and daily e-cigarette use for ≥4 years.
5. Dual-user group: participants who reported daily use of both cigarettes and e-cigarettes with a history of cigarette use ≥4 pack years and vaping for ≥4 years.

Participants were excluded if they had a history of unstable cardiopulmonary disease, such as uncontrolled hypertension, arrhythmias, ischemic heart disease, or heart failure. Individuals with any diagnosed acute or chronic respiratory conditions—including asthma, COPD, interstitial lung disease, pulmonary fibrosis, or respiratory infection within the previous 4 weeks, were also excluded. Additional exclusion criteria included current or past use of respiratory medications such as inhaled or systemic corticosteroids, bronchodilators, or leukotriene modifiers; neuromuscular disorders that could impair pulmonary function or exercise capacity (e.g., muscular dystrophy, myasthenia gravis); prior thoracic surgery or congenital lung abnormalities; and the use of inhaled substances other than cigarettes or e-cigarettes, such as shisha, or recreational inhalants.

All assessments were conducted in the same facility, with a standardized 30-min rest period between tests to minimize fatigue and ensure reliable measurements. A BMC College Clinic physician was available on site throughout the study to provide immediate medical support in the event of any emergencies.

### Pulmonary function test

All participants underwent spirometry to evaluate pulmonary function, specifically measuring Forced Vital Capacity (FVC), Forced Expiratory Volume in the first second (FEV_1_), and the FEV_1_/FVC ratio. The results were reported in liters (L) and as percentages of predicted normal values (% predicted). Spirometry was conducted using a MasterScreen PFT system powered by SentrySuite Software (Vyaire Medical), adhering to the guidelines of the European Respiratory Society (ERS), and the American Thoracic Society (ATS) (27).

The participants were seated and wore a nose clip during the test. Following demonstration and coaching, participants executed a maximal inspiration to total lung capacity, succeeded by a forceful and rapid exhalation through a disposable mouthpiece until no further air could be expelled, with a minimum exhalation duration of 6 s followed by a rapid inspiration.

#### Six minute walk test

Participants underwent a six-minute walk test conducted in accordance with the American Thoracic Society (ATS) guidelines (28). The test was administered along a flat, indoor corridor measuring 30 m, with cones marking intervals of 1–3 m at turnaround points. Following the confirmation of eligibility and baseline vital signs, participants were instructed to walk as far as possible within a 6-min duration at a self-selected pace, explicitly avoiding running. Participants were permitted to decelerate or rest as necessary and were provided with time prompts at 1-min intervals. The timing commenced with the participant's first step and continued through any rest periods. Upon completion of the 6 min, participants ceased walking, and the total distance was calculated based on the number of completed laps plus any final partial lap. Heart rate, SpO_2_, and Borg dyspnea/fatigue scores were recorded both prior to and following the test.

#### Health-related quality of life

HRQoL was assessed using the RAND SF-36 questionnaire (29). It includes 36 items covering eight domains. Physical functioning (10 items), social functioning (two items), role limitation due to physical health problems (four items), role limitation due to emotional problems (four items), bodily pain (two items), emotional wellbeing (five items), general health perceptions (five items), and energy/fatigue (four items). A higher score indicated a healthier state, with scores for each domain ranging from zero (worst) to 100 (best).

### Sample size

*A priori* power analysis was conducted using G^*^Power version 3.1.9.7 (30). Assuming a medium effect size (*f* = 0.25), an alpha level of 0.05, and 80% power, the estimated total sample size for a one-way ANOVA comparing five groups was approximately 200 participants (around 40 per group).

### Ethical approval

This study was approved by the Institutional Ethical Committee at BMC (Approval No. RES 2022-0036). All procedures followed the ethical standards of the committee and the principles of the Declaration of Helsinki. Written informed consent was obtained from all participants prior to enrollment. Participation was voluntary, and individuals retained the right to withdraw at any stage. Confidentiality was maintained throughout, with all data anonymized and securely stored to ensure privacy and data protection

### Statistical analysis

The Shapiro–Wilk test was used to assess normality. As the data were not normally distributed, continuous variables were presented as median and interquartile range (IQR), while categorical variables were summarized as frequencies and percentages (%). Group differences in continuous variables were analyzed using the Kruskal–Wallis test, with effect sizes reported as eta-squared (*η*^2^). *Post-hoc* pairwise comparisons were performed using Dunn's test with Bonferroni correction and adjusted *p*-value (Adj.*p*) were reported. A *p*-value ≤ 0.05 was considered statistically significant. Data analysis was conducted using SPSS software (version 25.0, IBM Corp., Armonk, NY, USA).

## Results

### Study population characteristics

**Figure 1:**
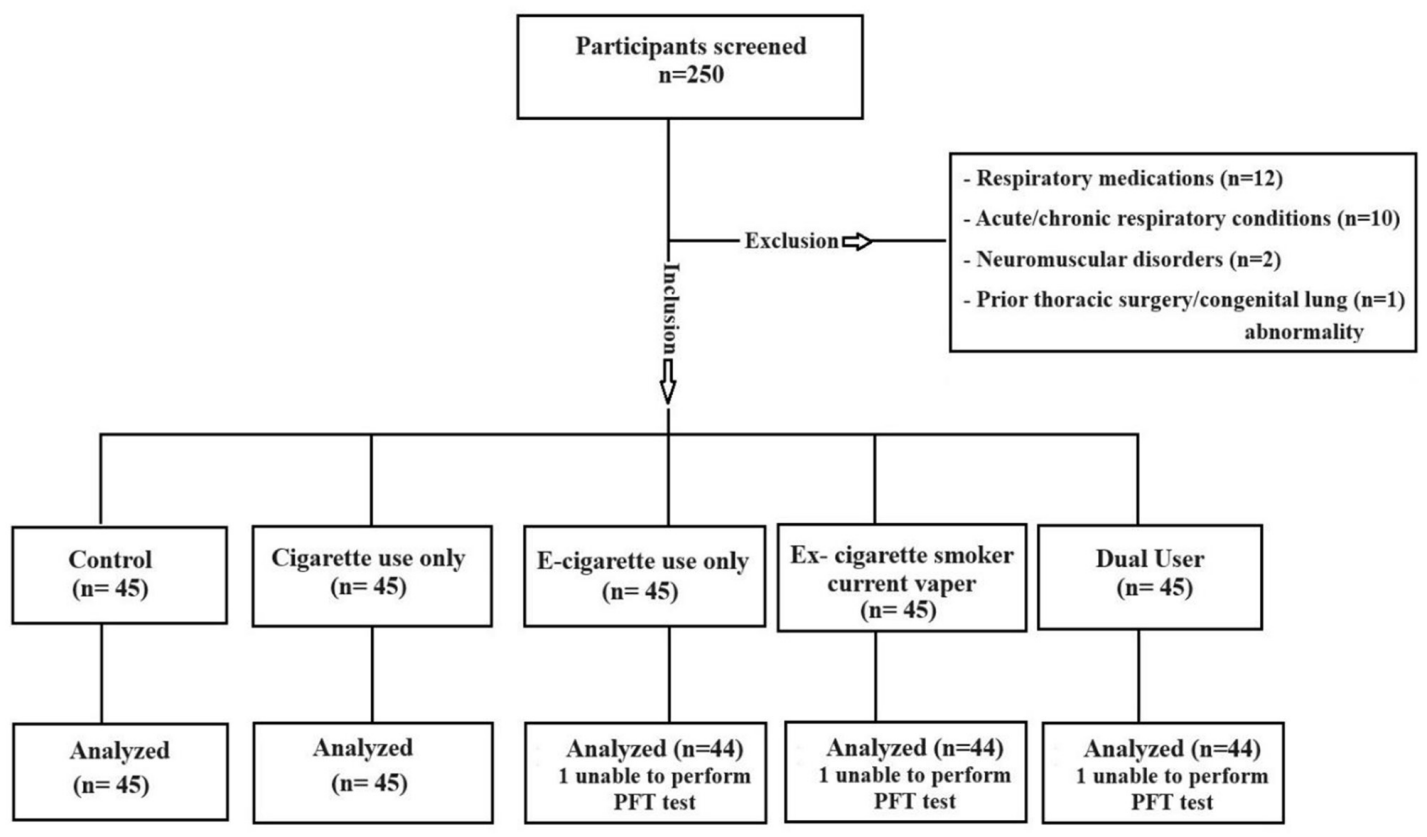
Flowchart of data collection.

A total of 250 individuals were screened for eligibility, of whom 28 were excluded (including those not meeting inclusion criteria and three participants who were unable to perform pulmonary function tests). Consequently, 222 participants were enrolled and analyzed across the five study groups (Figure 1). No significant differences were observed in age or BMI across groups. However, a significant difference in gender distribution was found among the groups (*p* = 0.007), with male participants comprising the majority in all groups (Table 1). The median number of cigarettes smoked per day was 20 (IQR 10) among cigarette-only users and dual users. The median smoking duration was 5 pack years (IQR 6) in cigarette-only users, 5.5 pack years (IQR 4) in ex-cigarette smoker current vapers, and 7 pack years (IQR 5) in dual users. The median duration of e-cigarette use was 5 years (IQR 1) in e-cigarette–only users, 4 years (IQR 1) in ex-cigarette smoker current vapers, and 4 years (IQR 2) in dual users; the corresponding median daily e-cigarette puffs per day were 150 (IQR 80), 180 (IQR 100), and 195 (IQR 140) respectively.

**Table 1:** Socio-demographic data (*n* = 222).

**Parameters**		**Group 1 Control (*n* = 45)**	**Group 2 Cigarette use only (*n* = 45)**	**Group 3 E-cigarette use only (*n* = 44)**	**Group 4 Ex-cigarette smoker current vaper (*n* = 44)**	**Group 5 Dual user (*n* = 44)**	***p*** **-value**
Gender *n* (%)	Male	35 (77.8)	42 (93.33)	33 (75)	42 (95.45)	41 (93.18)	0.007
	Female	10 (22.2)	3 (6.67)	11 (25)	2 (4.55)	3 (6.82)	
Age (years) Median (IQR)		29 (7)	30 (9)	27 (5)	27 (5)	28 (6)	0.06
BMI Kg/m^2^ Median (IQR)		23.91 (4.23)	25.15 (5.03)	23.30 (7)	23.91 (6.02)	23.75 (4.58)	0.83

### Pulmonary function test and 6MWT

Spirometry revealed mild to moderate obstructive patterns among cigarette-only users and dual users. FEV_1_ (L) was significantly reduced in cigarette-only users, ex-cigarette smoker current vapers, and dual users compared with controls (*H* = 35.36, df = 4, *p* < 0.001, *η*^2^ = 0.144, indicating a large effect). Similar reductions were observed for FEV_1_ (% predicted) across groups (*H* = 80.69, df = 4, *p* < 0.001, *η*^2^ = 0.35, also indicating a large effect), with dual users exhibiting the lowest values. FVC (L) did not differ significantly across groups (*p* = 0.24), however, FVC (% predicted) was modestly but significantly lower in smokers and dual users compared with controls (*H* = 17.84, df = 4, *p* = 0.001, *η*^2^ = 0.064, indicating a medium effect). The FEV_1_/FVC ratio was markedly reduced in cigarette-only users, ex-cigarette smoker current vapers, and dual users, with the greatest reduction observed among dual users (*H* = 66.54, df = 4, *p* < 0.001, *η*^2^ = 0.288, indicating a large effect; Table 2).

**Table 2:** Pulmonary function test outcomes among control participants, cigarette users, E-cigarette users, ex-smokers currently vaping, and dual users (*n* = 222).

**Parameters**	**Group 1 Control (*n* = 45)**	**Group 2 Cigarette use only (*n* = 45)**	**Group 3 E-cigarette use only (*n* = 44)**	**Group 4 Ex-cigarette smoker current vaper (*n* = 44)**	**Group 5 Dual user (*n* = 44)**	***H*** **statistics**	***p*** **-value**	****η* ^2^***
FEV_1_ (L)	3.7 (0.93)	3.21 (0.84)	3.34 (0.83)	3.31 (0.92)	3.07 (0.54)	35.36	<0.001	0.144
FEV_1_ (% predicted)	93 (12)	78 (15)	85 (15)	80 (15)	75 (9)	80.69	<0.001	0.35
FVC (L)	4.53 (1.09)	4.06 (1.07)	4.33 (1.51)	4.19 (0.85)	4.11 (1.28)	–	0.24	–
FVC (% predicted)	92 (15)	84 (15)	92 (20.5)	89 (14.5)	87 (14.75)	17.84	0.001	0.064
FEV_1_/FVC (%)	91 (11.05)	81 (12)	92.12 (15.9)	77 (17.75)	71.5 (16.25)	66.54	<0.001	0.288

Values are presented as Median (IQR).

**Figure 2:**
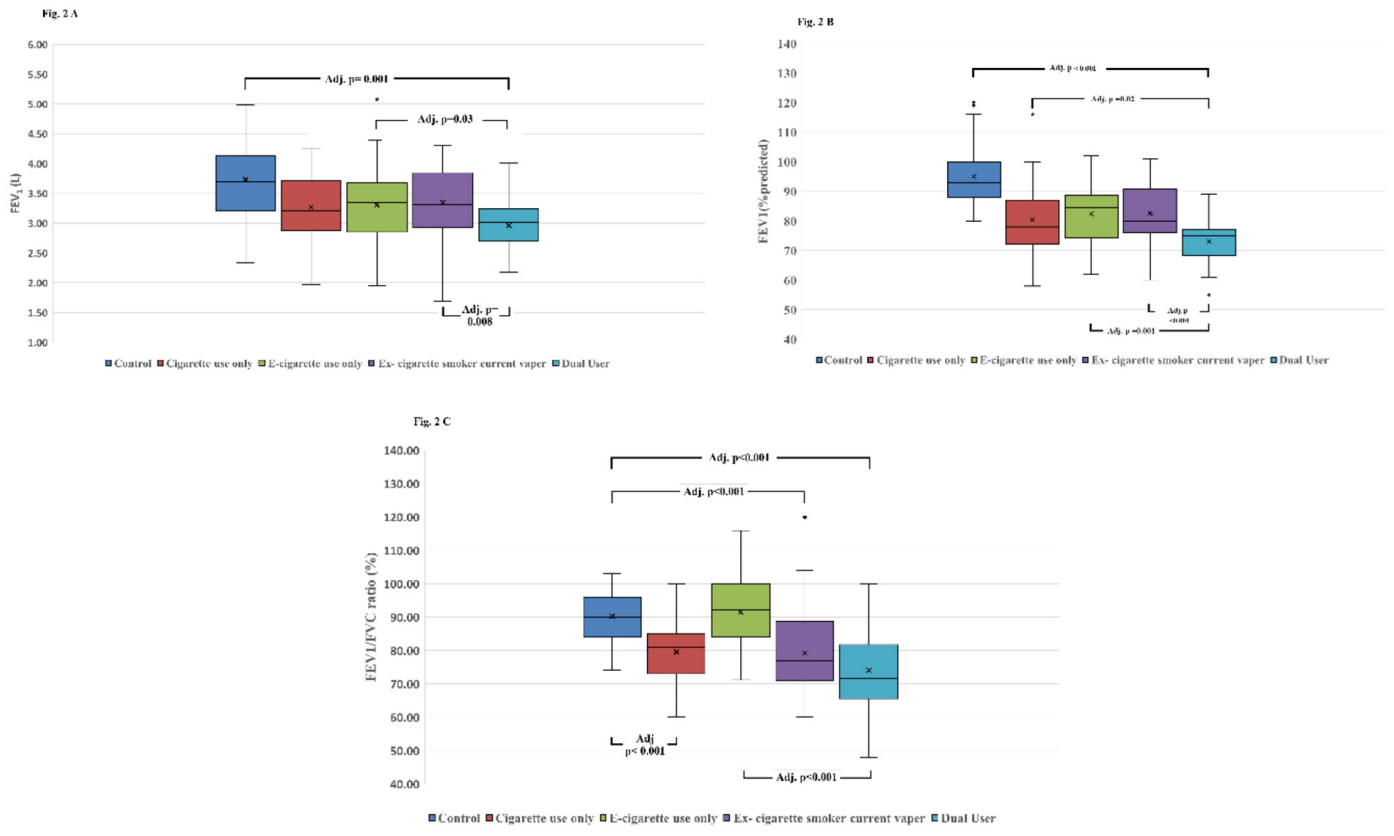
Comparison of pulmonary function test between the groups. **(A)** FEV_1_ (L), **(B)** FEV_1_ (% predicted), and **(C)** FEV_1_/FVC ratio.

*Post-hoc* pairwise comparisons (Dunn's test with Bonferroni correction) showed that the dual user group had significantly lower FEV_1_ (L) compared with, the control group (Adj.*p* < 0.001), the e-cigarette–only group (Adj.*p* = 0.03), and the ex-cigarette smoker current vaper group (Adj.*p* = 0.008; Figure 2A). Similarly, FEV_1_ (% predicted) was lower in dual users compared with all other groups: controls (*p* < 0.001), cigarette-only users (Adj.*p* = 0.02), e-cigarette–only users (Adj.*p* = 0.001), and ex-cigarette smoker current vapers (Adj.*p* < 0.001; Figure 2B). For the FEV_1_/FVC ratio, dual users showed significantly lower values compared with controls and e-cigarette–only users (both Adj.*p* < 0.001). Comparable reductions were also observed among cigarette-only users and ex- cigarette smoker current vapers when compared with controls and e-cigarette–only users (both Adj.*p* < 0.001; Figure 2C). The 6MWT showed no significant differences between groups for walking distance, dyspnea scores, SpO_2_, and heart rate (Table 3).

**Table 3:** Six-minute walk distance and physiological responses in control participants, cigarette users, E-cigarette users, ex-smokers currently vaping, and dual users (*n* = 222).

**Parameters**	**Group 1 Control (*n* = 45)**	**Group 2 Cigarette use only (*n* = 45)**	**Group 3 E-cigarette use only (*n* = 44)**	**Group 4 Ex-cigarette smoker current vaper (*n* = 44)**	**Group 5 Dual user (*n* = 44)**	***p*** **-value**
Walked distance (m)	709.68 (60.19)	697.44 (84.65)	700.61 (87.85)	708.55 (78.70)	700.81 (84.24)	0.18
Dyspnea score post test	0 (0)	0 (0)	0 (0)	0 (0)	0 (0)	0.48
Baseline SpO_2_	100 (1)	99 (2)	100 (1)	100 (1)	99 (1.75)	0.14
Post test SpO_2_	100 (1)	100 (1)	100 (1)	100 (1)	99.5 (1)	0.92
Baseline HR	84 (19)	83 (19)	81 (20)	86 (28)	86.5 (20)	0.86
Post test HR	103 (24)	99 (36)	106.5 (28)	100.5 (22)	103.5 (31)	0.87

Values are presented as Median (IQR).

### Health-related quality of life characteristics

The SF-36 questionnaire findings showed significant reductions in several domains across groups. Physical functioning was significantly lower (*H* = 35.11, df = 4, *p* < 0.001, *η*^2^ = 0.14) and social functioning (*H* = 35.11, df = 4, *p* < 0.001, *η*^2^ = 0.14) both demonstrating large effect sizes. Emotional wellbeing was also reduced (*H* = 19.51, df = 4, *p* = 0.001, *η*^2^ = 0.07) with a medium effect, whereas role limitations due to physical health showed a smaller but significant reduction (*H* = 10.33, df = 4, *p* = 0.03, *η*^2^ = 0.02). Dual users and cigarette-only users generally reported the lowest scores compared with controls, indicating reduced health-related quality of life. No significant differences were observed for role limitations due to emotional problems, energy/fatigue, pain, general health, or health change. In Table 4, the groups across the domains of the questionnaire were reported.

**Table 4:** Comparison of health-related quality of life measures among control participants, cigarette users, E-cigarette users, ex-smokers currently vaping, and dual users (*n* = 222).

**Parameters**	**Group 1 Control (*n* = 45)**	**Group 2 Cigarette use only (*n* = 45)**	**Group 3 E-cigarette use only (*n* = 44)**	**Group 4 Ex-cigarette smoker current vaper (*n* = 44)**	**Group 5 Dual user (*n* = 44)**	***p*** **-value**
Physical functioning	100 (10)	85 (20)	90 (20)	90 (15)	90 (10)	<0.001
Role limitations due to physical health	95 (12.5)	100 (25)	92.5 (25)	100 (25)	80 (25)	0.03
Role limitation due to emotional problem	90 (20)	100 (30)	97.5 (28.75)	95 (27.5)	80 (30)	0.36
Energy fatigue	80 (15)	80 (20)	80 (23.75)	75 (15)	80 (13.45)	0.12
Emotional wellbeing	90 (20)	80 (23)	80 (14.38)	80 (16.25)	78 (18)	0.001
Social function	87.5 (12.5)	80 (27.50)	80 (23.75)	87.5 (27.5)	75 (16.88)	<0.001
Pain	90 (15)	90 (22.50)	97.50 (18.75)	85 (22.50)	90 (20)	0.09
General health	85 (10)	80 (12.5)	80 (23.75)	80 (20)	80 (17.5)	0.08
Health change	80 (25)	75 (30)	77.5 (13.75)	75 (25)	77.5 (10)	0.39

Values are presented as Median (IQR).

The *post-hoc* analysis revealed significant group differences across several SF-36 domains. In the physical functioning domain, dual users had lower scores compared with controls (Adj.*p* < 0.001), cigarette-only users were lower than controls (Adj.*p* < 0.001), and ex-cigarette smoker current vapers were lower than controls (Adj.*p* = 0.04). For role limitations due to physical health, dual users scored significantly lower than e-cigarette–only users and ex-cigarette smoker current vapers (Adj.*p* = 0.049). In the emotional wellbeing domain, all groups showed reductions compared with controls: dual users (Adj.*p* = 0.003), cigarette-only users (Adj.*p* = 0.01), ex-cigarette smoker current vapers (Adj.*p* = 0.001), and e-cigarette–only users (Adj.*p* = 0.04). In social functioning, dual users had significantly lower scores compared with controls (Adj.*p* < 0.001), cigarette-only users (Adj.*p* = 0.006), e-cigarette–only users (Adj.*p* < 0.001), and ex-cigarette smoker current vapers (Adj.*p* = 0.002).

## Discussion

The present investigation demonstrated clinically significant impairments in lung function and quality of life among young adults using cigarettes, with dual users showing the greatest decline. Both cigarette and dual users exhibited reduced FEV_1_ and FEV_1_/FVC ratios, accompanied by lower scores in physical functioning, role limitations due to physical health, emotional wellbeing, and social functioning compared with controls. Ex-smokers who currently vape showed similar patterns, whereas e-cigarette–only users displayed intermediate values without clinically significant reductions, suggesting that vaping alone may be less harmful than smoking but still associated with adverse respiratory and psychosocial outcomes.

The cumulative exposure to cigarette smoking over time contributes to a disproportionate decline in FEV_1_ relative to FVC, resulting in a reduced FEV_1_/FVC ratio (31, 32). Similarly, our study identified a significant decrease in FEV_1_, FEV_1_% predicted, and FEV_1_/FVC among current smokers. These physiological impairments align with the early trajectory of obstructive lung disease and highlight the measurable impact of smoking even in relatively young or asymptomatic populations (33).

Current evidence concerning the long-term impact of vaping on pulmonary function remains both limited and evolving. In a 3.5-year prospective observational study, Polosa et al. reported that individuals who exclusively used e-cigarettes daily exhibited no significant alterations in lung function when compared to never-smokers (34). Additionally, these individuals showed no pathological abnormalities on high-resolution computed tomography (HRCT) and did not consistently present respiratory symptoms. Conversely, Digambiro et al. identified significantly reduced forced expiratory volume in FEV_1_, FVC, and FEV_1_/FVC ratios in young adult e-cigarette users compared to non-smokers (35). However, the study did not specify whether the e-cigarette users were former or concurrent cigarette smokers, thereby limiting the interpretability of these findings. In this study, exclusive e-cigarette users showed higher FEV_1_, FEV_1_ % predicted, and FEV_1_/FVC ratios than cigarette-only and dual users, with FVC comparable to controls, indicating minimal pulmonary impairment. Additionally, ex-smokers who vape demonstrated intermediate lung function and quality of life scores, suggesting partial mitigation of prior smoking effects. These findings support the potential of exclusive e-cigarette use as a harm-reduction strategy, consistent with previous studies, while highlighting the need for further research on long-term safety and clinical outcomes (36–38).

Nicotine and chemical inhalation from cigarettes and e-cigarettes impairs oxygen uptake and utilization, reducing exercise capacity (39, 40). Dinkelo et al. found physical training was highest in never users, followed by vapers, smokers, and dual users (41). However, in the current study there were no significant differences in 6-MWT performance, possibly due to the young age and submaximal nature of testing. Reduced cardiopulmonary fitness from cigarette and e-cigarette use affects daily activities and thereby quality of life (12, 42). Physical functioning and role limitations due to physical health were significantly reduced among cigarette users, ex-smokers who vape, and dual users in our study. Emotional wellbeing and social function declined among user groups, though these outcomes may be influenced by various psychosocial factors like mental health conditions, social support, and stress (43, 44). Nevertheless, these variables were not assessed in our study.

Dual users, who often initiate e-cigarette use as a perceived safer alternative or to aid smoking cessation. However, people who use e-cigarettes end up using both possibly due to either higher nicotine dependence or dissatisfaction with lower levels of nicotine in the vape (45–47). In the current analysis, greatest impairments were observed in this group, FEV_1_ (% predicted) declined by 18.5% and FEV_1_/FVC by 21.42% compared with controls and modest FVC reductions, indicating predominantly obstructive changes. HRQoL was also most affected. These findings suggest that combined exposure to cigarettes and e-cigarettes may have additive or synergistic effects on respiratory and psychosocial health, highlighting a critical gap in long-term dual-use research.

This study has several limitations, including a predominantly male sample, a cross-sectional design precluding causal inference, and unmeasured potential confounders such as lifestyle, socioeconomic, and psychosocial factors. This study did not evaluate other potential sources of exposure, such as secondhand smoke, occupational pollutants, or environmental factors (e.g., biomass or charcoal exposure), which may contribute to residual confounding. Additionally, inflammatory and oxidative stress biomarkers were not assessed, limiting insights into systemic effects of cigarette and e-cigarette use.

Future longitudinal studies with larger, more diverse populations are needed to establish causality and improve generalizability. Adjusting for confounders and incorporating inflammatory and oxidative stress biomarkers would provide deeper insights into the systemic effects of cigarette and e-cigarette use.

## Conclusion

This study observed that young adults who use cigarettes, e-cigarettes, and use of both exhibit varying degrees of impairment in lung function and HRQoL compared to those who have never used these products. Among the groups, daily dual users demonstrated the most significant deficits in respiratory and psychosocial health, whereas former smokers who currently use e-cigarettes and exclusive e-cigarette users exhibited intermediate and relatively better outcomes, respectively. These patterns represent associations observed in this cross-sectional sample and should not be construed as evidence of causality or reduced harm. Furthermore, the predominance of younger adult male participants limits the generalizability of the findings. Nonetheless, these results highlight the necessity for ongoing tobacco prevention efforts and public health education targeting all forms of tobacco and nicotine use, particularly the combined use of products, to enhance respiratory health and wellbeing among young adults.

## Data Availability

The raw data supporting the conclusions of this article will be made available by the authors, without undue reservation.
