# Supplementary figures and images for: Comparative effects of cigarette smoking, e-cigarette use, and dual use on pulmonary function, exercise capacity, and quality of life in young adults: a cross-sectional study
Source: Front Public Health. 2026 Jan 14;13:1707230. doi: 10.3389/fpubh.2025.1707230 (PMC12847274; doi:10.3389/fpubh.2025.1707230)

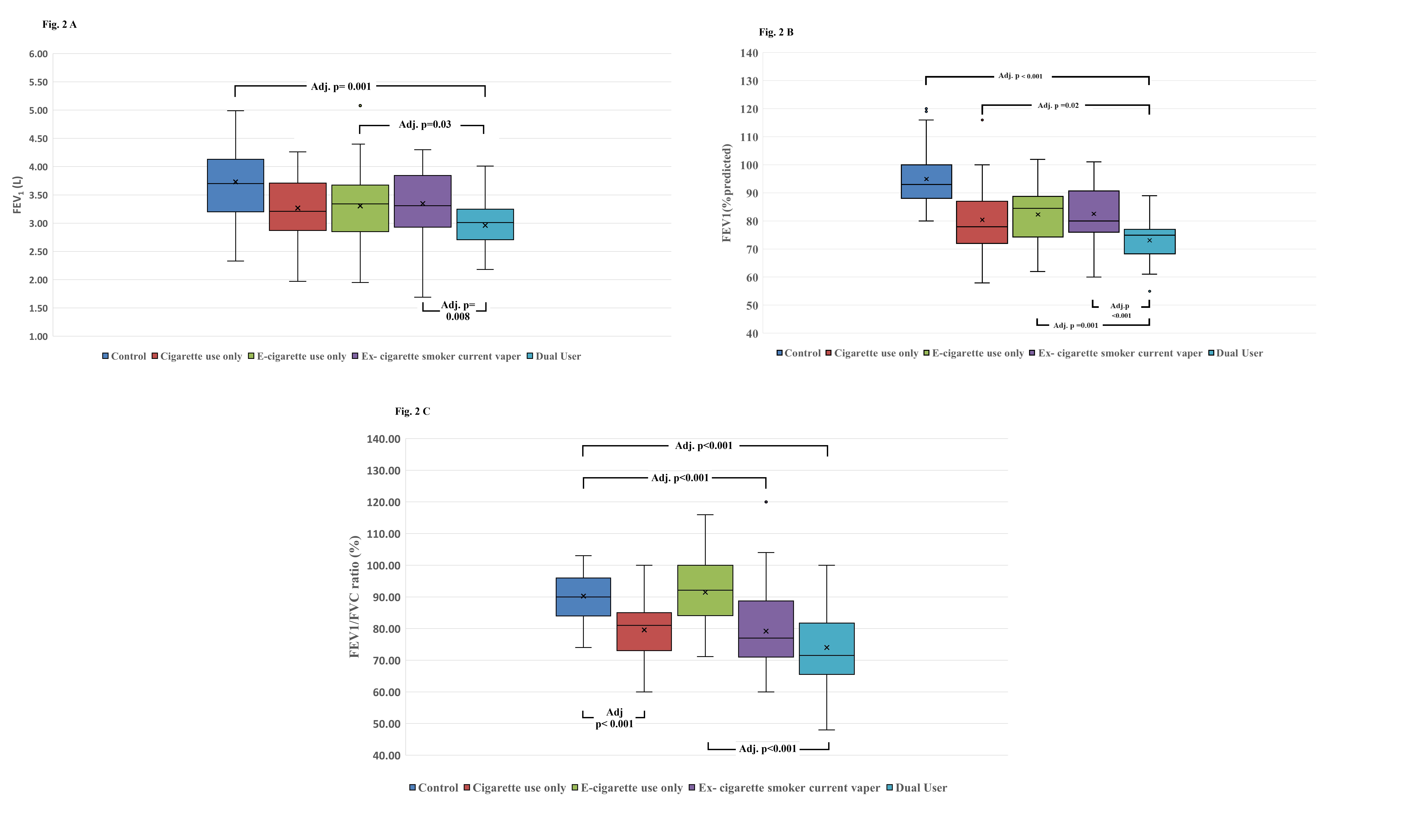

Supplement: Supplementary file 1 [file Image_1.png]
